# Long non-coding RNA MEG3 inhibits cervical cancer cell growth by promoting degradation of P-STAT3 protein via ubiquitination

**DOI:** 10.1186/s12935-019-0893-z

**Published:** 2019-07-08

**Authors:** Jun Zhang, Yali Gao

**Affiliations:** 1 Department of Obstetrics and Gynecology, The Second Clinical Medical College (Shenzhen People’s Hospital), Jinan University, Shenzhen, 518020 People’s Republic of China; 2 Department of Ophthalmology, The Second Clinical Medical College (Shenzhen People’s Hospital), Jinan University, Shenzhen, 518020 People’s Republic of China

**Keywords:** Cervical cancer, LncRNA, MEG3, STAT3

## Abstract

**Background:**

Maternally expressed 3 (MEG3) plays an important role in cervical cancer development, but its exact role remains unclear. Here, we explored the specific regulatory mechanism of MEG3 and its downstream proteins in cervical cancer cells.

**Methods:**

The effect of MEG3 on tumor formation ability of cervical cancer cells was determined in nude mice. The direct binding of MEG3 to phosphorylated signal transducer and activator of transcription 3 (P-STAT3) was detected by RNA pull-down and RNA-binding protein immunoprecipitation (RIP) assays. Cycloheximide (CHX)-chase and ubiquitination assays were performed to determine the regulatory effect of MEG3 on P-STAT3 ubiquitination. Clone formation assay and flow cytometry were used to evaluate the effect of the MEG3-STAT3 regulatory axis on cell proliferation and apoptosis.

**Results:**

In vivo tumor formation experiments showed that MEG3 inhibited the tumor formation ability of cervical cancer cells. RNA pull-down and RIP assays demonstrated that MEG3 bound directly to P-STAT3 protein. CHX-chase and ubiquitination assay results showed that MEG3 promoted P-STAT3 degradation via ubiquitination. Clone formation assay and flow cytometry analysis results revealed that the inhibitory effect of MEG3 on P-STAT3 promoted apoptosis and inhibited proliferation of cervical cancer cells.

**Conclusion:**

MEG3 binds to P-STAT3 in cervical cancer cells, resulting in P-STAT3 ubiquitination and degradation and apoptosis and inhibition of proliferation of tumor cells. The in-depth elaboration of the MEG3-STAT3 regulatory axis in cervical cancer may clarify the mechanism of action of MEG3 and provide new ideas for cervical cancer treatment.

## Background

Cervical cancer is the fourth most common malignancy in women. According to the World Health Organization (WHO) data, 530,000 new cases of cervical cancer are reported every year, and about 250,000 women die from cervical cancer worldwide; of these, 80% patients belong to developing countries. In China, there are about 140,000 new cases of cervical cancer every year, and about 37,000 deaths are reported [1]. Therefore, there is an urgent need to explore the pathogenesis of cervical cancer.

Maternally expressed 3 (MEG3) gene is a long non-coding RNA that regulates gene expression and has been shown to have tumor suppressive effects in breast cancer, gallbladder cancer, and retinoblastoma [2–4]. Our previous studies have revealed the decrease in MEG3 expression in cervical cancer tissues and its close association with the prognosis of patients. Upregulation in MEG3 expression was shown to inhibit the proliferation of cervical cancer cells and promote apoptosis, while MEG3 downregulation could promote the proliferation of cervical cancer cells and inhibit their apoptosis. The hypermethylation of MEG3 gene promoter may result in low expression of MEG3 in cervical cancer, eventually leading to the proliferation of malignant cells and decrease in cell apoptosis [5–7]. Therefore, the abnormality of the regulatory network of MEG3 gene expression is closely related to the occurrence and development of cervical cancer. However, the regulatory proteins acting downstream of MEG3 in cervical cancer cells are unknown, demanding further investigation.

To investigate the specific mechanism of action of MEG3 in cervical cancer, we evaluated the effect of MEG3 on in vivo tumor formation ability of cervical cancer cells through animal experiments and preliminarily clarified the mechanism of the interaction between MEG3 and phosphorylated signal transducer and activator of transcription 3 (P-STAT3) protein by RNA pull-down, RNA-binding protein immunoprecipitation (RIP), cycloheximide (CHX)-chase, and ubiquitination assays.

## Materials and methods

### Samples

A total of 22 paired cervical cancer tissues and adjacent normal tissues were collected from patients who had undergone surgery between April 2016 and September 2016. Patients did not receive any preoperative cancer treatments, such as radiotherapy or chemotherapy. All tissues were evaluated by two pathologists and was frozen in liquid until use. Informed consent was obtained from all participating subjects and the study was approved by the Ethics Committee of Shenzhen People’s Hospital.

### Establishment of cervical cancer cells stably expressing high or low level of MEG3

A lentiviral vector carrying MEG3 (MEG3 group) and its control lentiviral vector (vector group), as well as a lentiviral vector carrying MEG3-specific short-hairpin RNA (shRNA; MEG3 shRNA group) and its control lentiviral vector (NC shRNA group) were purchased from Shanghai GenePharma Co. Ltd. Cell transfection was performed according to the lentiviral protocol. Siha or HeLa cells in logarithmic growth phase were seeded into 12-well plates at a density of 0.5 × 10^5^ cells/well, and 40% confluent cells were transfected with different groups of lentiviral vectors. After screening with puromycin (1 μg/mL), cervical cancer cells stably expressing high or low level of MEG3 were obtained.

### In vivo tumor formation

Twenty female NOD/SCID mice, 4–5 weeks old, were purchased from the animal center of Sun Yat-sen University and raised under specific pathogen-free (SPF) conditions. Cells from MEG3, vector, MEG3 shRNA, and NC shRNA groups were washed with phosphate-buffered saline (PBS) and resuspended at a density of 1 × 10^6^ cells/mL. Mice were randomly divided into four groups and subcutaneously injected with 100 μL of cell suspension at their lower right flanks. Tumor size was measured every 4 days. All mice were sacrificed on day 21. Tumor tissues were collected and fixed with 10% paraformaldehyde. Paraffin sections of 3-μm thickness were obtained and stained and observed under an optical microscope. All animal experimental procedures were evaluated and approved by the Ethics Committee of Shenzhen People’s Hospital.

### Cell culture, transient transfection, real-time quantitative polymerase chain reaction (RT-qPCR), western blotting, cell cloning, and flow cytometry

Experimental procedures, reagents, and primers were the same as reported in our previous studies [5–7]. P-STAT3, STAT3, cleaved caspase-3 and c-MYC antibodies were purchased from Cell Signaling Technology (1:1000; Massachusetts, USA). The pcDNA-STAT3 plasmid mediating overexpression and its blank control pcDNA-NC and siRNAs against STAT3 (si-STAT3) and the nonsense control (si-NC) were all synthesized by GenePharma (Shanghai, China).

### Enzyme-Linked ImmunoSorbent Assay (ELISA)

P-STAT3 concentration in 22 samples of cervical cancer tissues and adjacent normal tissues was detected by P-STAT3 InstantOne ELISA™ kit (Thermo Scientific, Massachusetts, US), following the manufacturer’s instruction. Briefly, the protease inhibitor was added into the cervical cancer and adjacent normal tissues and then tissues were homogenized. After 30 min of 12,000 r/min centrifugation, total protein was extracted from the supernatant. The 50 mL of sample lysate, lysis mix and control lysate was added to microplate wells respectively. Then 100 μL of detection reagent was added to each assay well. After incubation for 20 min, the absorbance at 450 nm was measured using an ELISA microplate reader. The P-STAT3 level of cervical cancer tissue which relative to its pair adjacent normal tissue was calculated.

### STAT3 luciferase reporter assay

Siha and Hela cells were transfected with the P-STAT3-TA-luc plasmids (Beyotime Biotechnology, Shanghai, China) using the Lipofectamine 2000 (Invitrogen, California, US) following the manufacturer’s instruction. After transfection for 48 h, Firefly luciferase activities were assayed using the Luciferase Assay System (Promega, WI, USA) according to the manufacturer’s instructions.

### Preparation for immunofluorescence examination

Cells were washed with PBS and fixed with 4% paraformaldehyde at room temperature for 20 min, treated with 0.5% Triton X-100 for 20 min, and washed again with PBS before treatment with goat serum for 30 min at room temperature. The cells were overnight incubated with a primary antibody against P-STAT3 (1:200, CST) at 4 °C. Following incubation, the cells were washed with PBS and treated with a secondary antibody for 2 h in the dark, followed by staining with 4′,6-diamidino-2-phenylindole (DAPI) for 5 min at room temperature. Samples were rinsed with PBS and sealed with a mounting medium containing an anti-quenching agent.

### RNA pull-down assay

The RNA pull-down assay was performed using the Pierce™ Magnetic RNA–protein pull-down kit (Thermo, MA, USA). MEG3 and its antisense transcript were synthesized and purified in vitro and labeled with biotin. The experimental procedures were carried out according to the manufacturer’s instructions. Briefly, 3 μg of biotin-labeled RNA was mixed with 1 mg of protein extract, and the mixture was incubated with Dynabeads Myone Streptavidin T1 beads overnight at 4 °C. The RNA–protein complex was subjected to sodium dodecyl sulfate polyacrylamide gel electrophoresis (SDS-PAGE) and silver staining, followed by western blot analysis.

### RIP assay

We performed RIP assay using the Magna RNA-binding protein immunoprecipitation kit (Thermo, MA, USA). The experimental procedures were in accordance with the manufacturer’s instructions. Briefly, 3 μg of P-STAT3 and IgG control antibodies were overnight incubated with cell lysates at 4 °C. A total of 25 μL of protein A/G beads were incubated with the mixture for another 2 h. The co-precipitated RNAs were extracted for RT-qPCR and 2% agarose gel electrophoresis.

### Ubiquitination assay

Cells were transfected with ubiquitin (Ubbiotech, Changchun, China) and P-STAT3 plasmids using jetPRIME (Polyplus, Strasbourg, France). After 36 h of transfection, 20 μM of MG132 (Selleck Chemicals, Houston, TX, USA) was added to the medium for 4 h, followed by protein extraction for western blot analysis. Cell lysates were immunoprecipitated (IP) with the labeled antibodies and overnight incubated at 4 °C. The eluted proteins were determined by western blotting.

### CHX-chase assay

CHX-chase assay was performed using CHX (Selleck Chemicals), an inhibitor of protein synthesis. The cells in each group were mixed with 12.5 μg/mL of CHX and the expression of P-STAT3 protein was determined by western blot analysis at 0, 3, 6, 12, and 24 h.

### Statistical analysis

Statistical analysis of the data was performed using the SPSS 19.0 software. Measurement data were expressed as mean ± standard error of mean (mean ± SEM), and the difference was considered statistically significant at P < 0.05.

## Results

### Effect of MEG3 on the in vivo tumor formation ability of cervical cancer cells

**Fig. 1:**
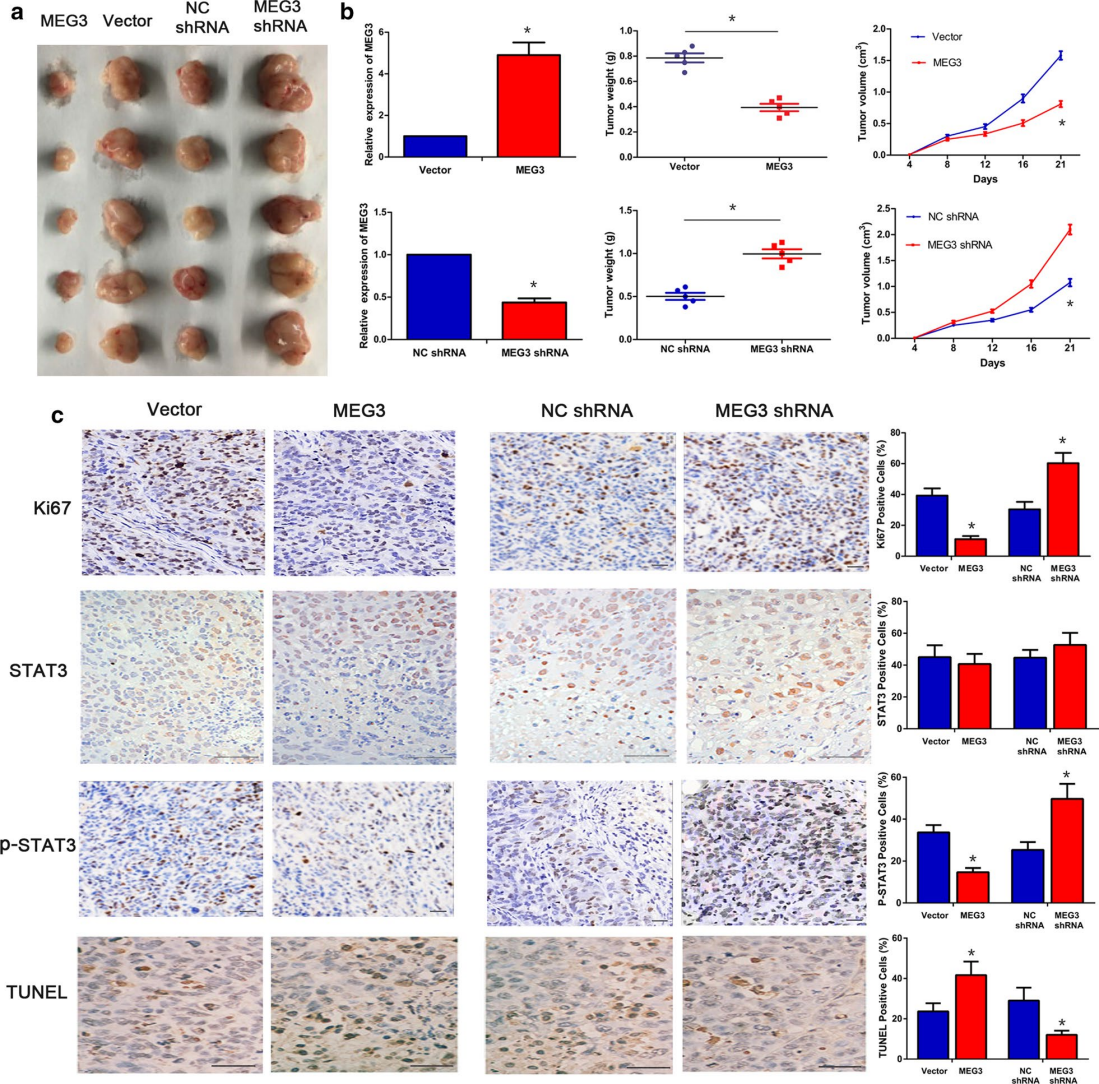
MEG3 inhibits the tumor formation ability of cervical cancer cells. The rate of tumor formation and tumor size in MEG3 group were significantly lower than those in the control group. In contrast, the tumor formation rate and tumor size were significantly higher in MEG3 shRNA group than in the control group (**a**, **b**). Immunohistochemistry examination of tumors showed that the expression of Ki67 and P-STAT3 proteins was significantly lower and the number of TUNEL-positive cells was significantly higher in MEG3 group than in the control group; the expression of Ki67 and P-STAT3 proteins was significantly higher and the number of TUNEL-positive cells was significantly lower in MEG3 shRNA group than in the control group. The difference of STAT3 expression was not significant. Scale bar = 50 μm (**c**). *P < 0.05

Mice were divided into four groups and subcutaneously injected with cervical cancer cells from the MEG3, vector, MEG3 shRNA, or NC shRNA group. The results showed that the tumor size was smaller and the tumor formation rate was slower in MEG3 group than in vector group. MEG3 shRNA group had larger subcutaneous tumors and faster rate of tumor formation than NC shRNA group. The difference was statistically significant (Fig. 1a, b, P < 0.05).

The tumor tissues were collected for RT-qPCR and MEG3 expression was confirmed to be high in MEG3 group and low in MEG3 shRNA group (Fig. 1b, P < 0.05). The results of immunohistochemistry showed that the expression of P-STAT3 and Ki-67 proteins significantly decreased and the rate of terminal deoxynucleotidyl transferase dUTP nick end labeling (TUNEL)-positive cells significantly increased in MEG3 group than in the control group. On the contrary, the expression of P-STAT3 and Ki-67 proteins significantly increased and the number of TUNEL-positive cells significantly decreased in MEG3 shRNA group (Fig. 1c, P < 0.05). The difference was statistically significant. However, there was no significant difference in STAT3 expression between MEG3 group and the control group, or between MEG3 shRNA group and the control group (Fig. 1c, P > 0.05).

### Mutual regulation between MEG3 and P-STAT3

**Fig. 2:**
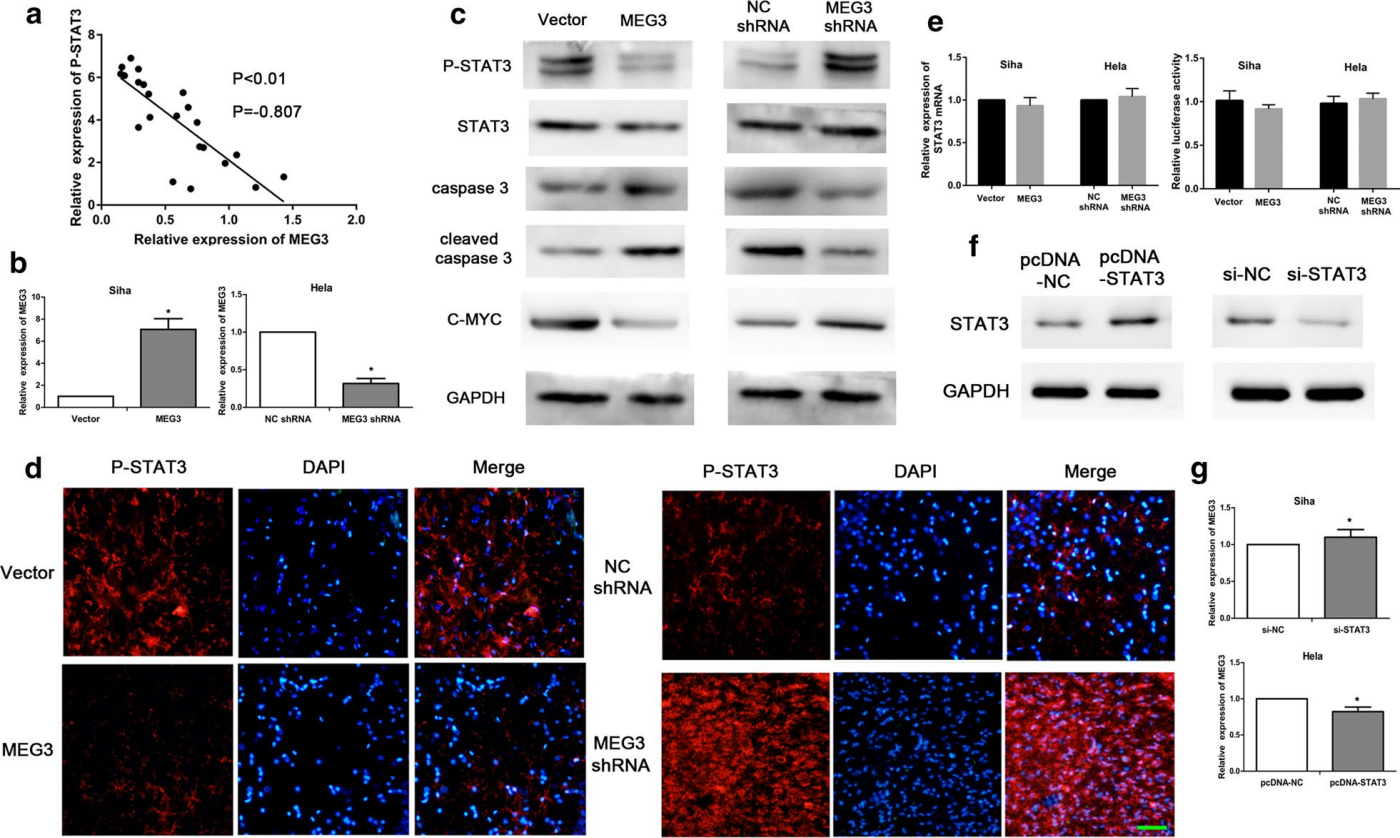
The mutual regulation between MEG3 and P-STAT3. The expression of P-STAT3 and MEG3 in cervical cancer tissues(n = 22)was detected by ELISA and RT-qPCR assays. Then Pearson correlation coefficient was calculated (**a**). RT-qPCR and western blot analysis results showed that the expression of P-STAT3 and c-MYC proteins decreased while that of caspase 3 and cleaved caspase 3 increased in Siha cells with high-level expression of MEG3. On the contrary, HeLa cells with low-level expression of MEG3 showed an increase in the expression of P-STAT3 and c-MYC proteins and a decrease in the expression of caspase 3 and cleaved caspase 3 (**b**, **c**). Immunofluorescence results confirmed that P-STAT3 protein was highly expressed in the cells from MEG3 group, while its expression was low in the cells from MEG3 shRNA group. Scale bar = 50 μm (**d**). RT-qPCR and luciferase assays showed that MEG3 had no significant effect on STAT3 gene expression (**e**). RT-qPCR and western blot analysis results revealed the absence of any significant change in the expression of MEG3 after upregulation or interference of STAT3 expression in cervical cancer cells (**f**, **g**). *P < 0.05

We first used ELISA and RT-qPCR to detect the relative expression of P-STAT3 and MEG3 in cervical cancer tissues. Then Pearson correlation analysis indicated that the expression of MEG3 was negatively correlated with that of P-STAT3 in cervical cancer tissues (Fig. 2a, P < 0.01, R = − 0.807). Next, we validated it in cervical cancer cell lines. The high-level expression of MEG3 in Siha cells from MEG3 group was confirmed by RT-qPCR. The expression of MEG3 in HeLa cells from MEG3 shRNA group was low, and the difference was statistically significant, indicative of the successful establishment of the stable cell lines following transfection (Fig. 2b, P < 0.05). The expression levels of STAT3, P-STAT3, caspase-3, cleaved caspase-3, and c-MYC were determined by western blot analysis. As a result, the expression of P-STAT3 and c-MYC was significantly lower in Siha cells from MEG3 group than Siha cells from the control group. On the other hand, the expression of caspase-3 and cleaved caspase-3 significantly increased in the cells from MEG3 group. In comparison with the control group of HeLa cells, MEG3 shRNA group of HeLa cells showed a significant decrease in the expression of P-STAT3 and c-MYC and a significant increase in the expression of caspase-3 and cleaved caspase-3 (Fig. 2c). However, the difference of STAT3 expression was not significant (Fig. 2c). The expression level of P-STAT3 protein was evaluated with immunofluorescence. The results showed that the fluorescence intensity was significantly lower in cells from MEG3 group than in cells from the control group. The fluorescence intensity from HeLa cells in MEG3 shRNA group was significantly increased as compared with that from the cells in the control group (Fig. 2d).

Furthermore, the expression level of P-STAT3 mRNA was evaluated with RT-qPCR. The results showed that the difference in STAT3 mRNA between MEG3 group and the control group, or between MEG3 shRNA group and the control group, was no significant (Fig. 2e, P > 0.05). Additionally, we evaluated the effect of MEG3 on STAT3 gene expression by luciferase assay. But we did not observed a significant difference of the luciferase signal in MEG3 group or in MEG3 shRNA group, compared to their control respectively (Fig. 2e, P > 0.05).

Moreover, the results of western blot analysis showed that STAT3 expression was high in HeLa cells transfected with pcDNA-STAT3 but low in Siha cells transfected with siRNA-STAT, indicative of the successful cell transfection (Fig. 2f). Then RT-qPCR was performed to determine the expression level of MEG3. The results revealed the absence of any significant difference in the expression of MEG3 between HeLa cells transfected with pcDNA-STAT3 and Siha cells transfected with siRNA-STAT3 (Fig. 2g, P > 0.05).

### MEG3 binds directly to P-STAT3

**Fig. 3:**
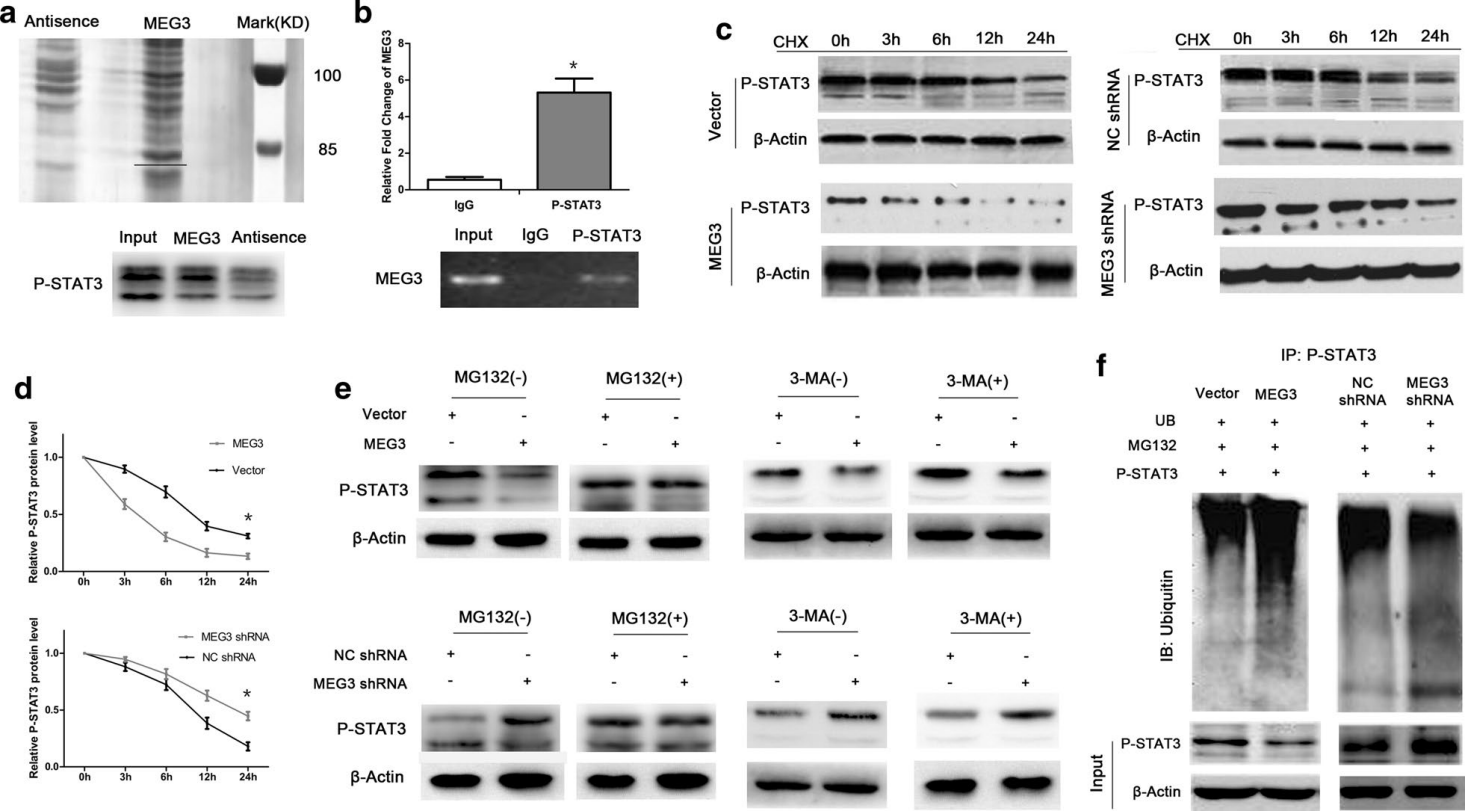
MEG3 promotes the degradation of P-STAT3 via ubiquitination. The RNA pull-down assay results suggest that MEG3 may bind to P-STAT3 protein (**a**). The results of RIP assay suggest that P-STAT3 protein may enrich MEG3 (**b**). CHX-chase assay results suggest that MEG3 may promote P-STAT3 protein degradation (**c**, **d**). All groups of cells were treated with 20 μM of an ubiquitination inhibitor MG132 for 4 h or 5 mM of an autophagy inhibitor 3-methyladenine (3-MA, Selleck Chemicals) for 2 h, followed by western blot analysis to determine changes in P-STAT3 expression (**e**). Ubiquitination assay was used to detect P-STAT3 ubiquitination (**f**) in all groups of cells. *P < 0.05

The bands specific to MEG3 were obtained by RNA pull-down assay and subjected to western blot analysis for protein identification. P-STAT3 protein level was significantly higher in MEG3 group than in the antisense group (Fig. 3a). Anti-P-STAT3 (IgG for control) was used for RIP assay. Both electrophoresis and RT-qPCR results of RIP products showed that anti-P-STAT3 could significantly enrich MEG3 (Fig. 3b, P < 0.05).

### MEG3 affects ubiquitination and degradation of P-STAT3 protein

The cells from MEG3, vector, MEG3 shRNA, and NC shRNA groups were treated with CHX, and the expression level of P-STAT3 protein was determined at 0, 3, 6, 12, and 24 h. The results showed that the degradation rate of P-STAT3 protein was significantly higher in MEG3 group than in the control group. The degradation rate of P-STAT3 protein significantly decreased in MEG3 shRNA group as compared with the control group. The difference was statistically significant (Fig. 3c, d, P < 0.05).

The cells from MEG3, vector, MEG3 shRNA, and NC shRNA groups were treated with MG132 or 3-MA. The difference in P-STAT3 protein expression was determined by western blot analysis. The results revealed the absence of any significant change in the expression of P-STAT3 protein between MEG3 + MG132 (+) group and vector + MG132 (+) group, suggesting that MG132 affected the regulatory effect of MEG3 on P-STAT3 protein. In comparison with vector + MG132 (+) group, MEG3 + 3-MA (+) group showed a significant increase in the expression of STAT3 protein, suggesting that 3-MA had no significant effect on the regulation of P-STAT3 protein by MEG3 (Fig. 3e).

Ubiquitination assay results suggested that P-STAT protein ubiquitination significantly increased in MEG3-overexpressing cells as compared with the control cells, whereas P-STAT protein ubiquitination significantly reduced in the cells exhibiting low expression of MEG3 (Fig. 3f).

### P-STAT3 protein is a functional protein downstream of MEG3

**Fig. 4:**
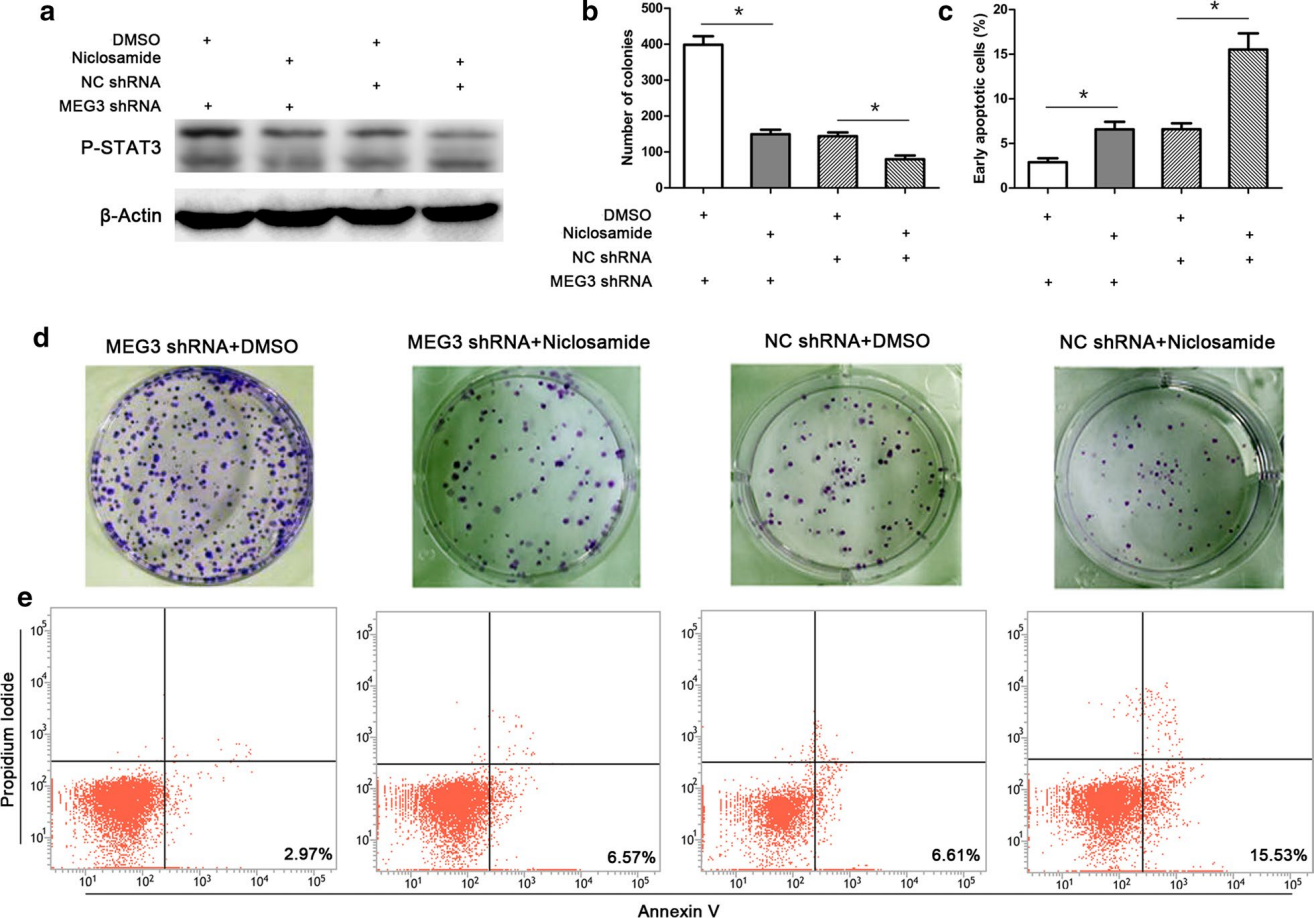
The effect of the MEG3-STAT3 regulatory axis on the proliferation and apoptosis of cervical cancer cells. Cells were treated with nifuroxazide (STAT3 phosphorylation inhibitor, Selleck Chemicals) at a final concentration of 20 mM in dimethyl sulfoxide (DMSO). Western blot analysis of P-STAT3 protein expression suggested that nifuroxazide may effectively decrease the level of P-STAT3 protein and reverse the regulation of P-STAT3 protein by MEG3 shRNA (**a**). The clone formation experiment results suggest that nifuroxazide may reverse the effect of MEG3 shRNA on the promotion of the proliferation of cervical cancer cells (**b**, **d**). Flow cytometry results indicate that nifuroxazide may reverse the effect of MEG3 shRNA on the inhibition of apoptosis of cervical cancer cells (**c**, **e**). *P < 0.05

To investigate whether the effects of MEG3 on the proliferation and apoptosis of cervical cancer cells are exerted through the regulation of P-STAT3 protein expression, cells were treated with niclosamide, an inhibitor of STAT3 protein phosphorylation. Cervical cancer cells were divided into MEG3 shRNA + dimethyl sulfoxide (DMSO) group, MEG3 shRNA + niclosamide group, NC shRNA + DMSO group, and NC shRNA + niclosamide group. The expression level of P-STAT3 protein was determined by western blot analysis. As a result, we found that the level of P-STAT3 protein significantly decreased in NC shRNA + niclosamide group as compared with NC shRNA + DMSO group, indicating that niclosamide effectively inhibited STAT3 phosphorylation (Fig. 4a). The level of P-STAT3 protein significantly decreased in MEG3 shRNA + niclosamide group as compared with MEG3 shRNA + DMSO group, suggesting that niclosamide could antagonize the stabilization of P-STAT3 protein by MEG3 shRNA (Fig. 4a).

The changes in the cellular functions were evaluated. Clone formation assay suggested that the number of clones formed by cells from MEG3 shRNA + niclosamide group was significantly lower than that formed by cells from MEG3 shRNA + DMSO group (Fig. 3b, d, P < 0.05). Flow cytometry results showed that the ratio of early apoptotic cells in MEG3 shRNA + niclosamide group was significantly higher than the ratio of apoptotic cells in MEG3 shRNA + DMSO group (Fig. 3c, e, P < 0.05). The difference was statistically significant.

## Discussion

We have previously demonstrated that MEG3 expression is low in cervical cancer tissues and is closely related to the prognosis of patients. In vitro study results have shown that the low expression of MEG3 may lead to the malignant proliferation of cervical cancer cells. The close correlation between MEG3 expression and cervical cancer was confirmed at tissue and cellular levels [5–7]. However, the inhibition of cervical cancer cell growth by MEG3 expression has not been validated in animal experiments, and its specific downstream proteins and mechanism of action are still unclear. Therefore, this study aimed to explore the mechanism of action of MEG3 in cervical cancer based on our previous studies.

We performed a tumor formation experiment in nude mice and found that MEG3 could significantly inhibit the growth of cervical cancer cells in nude mice. Immunohistochemistry (Ki67) and TUNEL assay results also confirmed that MEG3 expression was negatively correlated with the proliferative activity of tumor cells and positively correlated with the apoptosis of tumor cells. The above results indicate that MEG3 may inhibit the proliferation of cervical cancer cells in nude mice, and the results of animal experiments were consistent with our previous results of cellular experiments [5].

STATs are the downstream effectors of cytokine and growth factor receptors and bind to specific DNA promoters in the nucleus and regulate the expression of related genes [8]. Of the seven STAT family members in mammals, STAT3 has been identified as an oncogene. It is a transcription factor closely related to tumor proliferation, differentiation, apoptosis, invasion, and metastasis [9, 10]. For example, STAT-3 activation counteracts the effects of STAT-1 on p21 and p27 expression and activates a survival MAPK and AKT-dependent pathway which inhibits the occurrence of autophagy in pancreatic cancer cells [11]. Recent studies have confirmed the overexpression of STAT3 in cervical cancer tissues and its close relationship with human papillomavirus (HPV) infection, tumor metastasis, and poor prognosis of patients. STAT3 is an important oncogene in cervical cancer occurrence [12–14]. Although the specific mechanism of the aberrant activation of STAT3 is not well understood, it may be related to the abnormal regulation of STAT3 by non-coding RNAs [15, 16]. We analyzed the role of MEG3 and STAT3 in cervical cancer and found that these two proteins have overlapping functions in HPV infection and lymphatic metastasis. Therefore, whether there is a regulatory relationship between the MEG3 and STAT3 has aroused our interest.

We first analyzed whether MEG3 regulated STAT3 gene transcription by RT-qPCR. The results suggested that MEG3 had no significant effect on STAT3 transcription. Similarly, Western Blot and Luciferase assays also showed that MEG3 had no significant effect on the level of STAT3 protein. However, Western Blot indicated that MEG3 had regulatory effect on the P-STAT3 level. We also analyzed whether STAT3 protein regulates MEG3 transcription by RT-qPCR, and got negative results. These mean that MEG3 maybe only regulate the phosphorylation modification of STAT3 protein.

Based on this, we examined the expression of STAT3 and MEG3 in cervical cancer tissues and found a negative correlation between them. Then we examined the level of P-STAT3 protein in the tumor tissues in the tumor formation experiment and found that the expression of P-STAT3 protein was significantly decreased in MEG3 group. It was also confirmed that MEG3 could decrease the level of P-STAT3 protein in cervical cancer cell lines. These observations indicate that MEG3 may regulate P-STAT3 protein level and that P-STAT3 protein may act downstream of MEG3. We also analyzed whether P-STAT3 exerted feedback regulation of MEG3 and found that P-STAT3 had no obvious regulatory effect on MEG3 expression. Therefore, we performed the subsequent studies on the regulation of proteins by MEG3.

Studies have confirmed that MEG3 may directly bind to proteins and affect their stability [17]. Whether MEG3 directly binds to P-STAT3 protein to affect its stability is questionable. Through RNA pull-down and RIP assays, we confirmed that MEG3 could directly bind to P-STAT3 protein in cervical cancer cell lines and exert its regulatory effect. Whether the binding of MEG3 to P-STAT3 affect the stability of P-STAT3 protein (to achieve its regulatory function) was unknown. We treated all the groups of cells with CHX to block cell protein synthesis and determined P-STAT3 protein expression at 0, 3, 6, 12, and 24 h. The results showed that MEG3 could promote the degradation of P-STAT3 protein. To clarify the mechanism underlying MEG3-mediated degradation of P-STAT3, we treated cervical cancer cells with MG132 or 3-MA to block protein degradation via ubiquitination or autophagy, respectively, and performed western blot analysis to determine the difference in P-STAT3 expression among all the groups of cells. The results suggested that the ubiquitination inhibitor MG132 could significantly attenuate the degradation of P-STAT3 protein by MEG3, while the autophagy pathway inhibitor 3-MA had no significant effect on MEG3-mediated degradation of P-STAT3 protein. We performed ubiquitination assay to confirm that MEG3 could promote the ubiquitination of P-STAT3. The above experiments demonstrate that MEG3 may directly bind to P-STAT3 protein and promote its degradation via ubiquitination, thereby regulating its expression.

To determine whether the regulation of P-STAT3 protein by MEG3 affects the proliferation and apoptosis of cervical cancer cells, we reversed the regulation of P-STAT3 protein by MEG3 using a STAT3 protein phosphorylation inhibitor niclosamide. As a result, the promotion of proliferation and inhibition of apoptosis of cervical cancer cell lines by MEG3 shRNA were significantly attenuated through the antagonizing effect of niclosamide. These results suggest that MEG3 may exert its effect of inhibition of cell proliferation and promotion of cell apoptosis by affecting the stability of P-STAT3 protein.

In addition, accumulating evidence suggests the transcription level of c-Myc gene are drastically increased by JAK/STAT3 signal pathway. STAT3 can bind to C-MYC gene promoter and due to the active c-Myc overexpression [18–20]. Similarly, we found that MEG3 can regulate the level of P-STAT3 and c-Myc simultaneously in cervical cancer cells, indicating that MEG3 might regulate the expression of c-Myc through P-STAT3 indirectly. More importantly, recent studies suggest that c-Myc can be a promising therapeutic target molecule among Myc family in terms of the biological characteristics of cancer stem-like cells [18–20]. That means MEG3, as an upstream regulator of c-Myc, also has broad prospects in the biological treatment of cervical cancer.

## Conclusions

In conclusion, MEG3 may bind to P-STAT3 protein to promote its degradation via ubiquitination, thereby inhibiting cell proliferation and affecting the development of cervical cancer. The in-depth study of this mechanism of action may provide a new research direction for targeted therapy of cervical cancer.

## Data Availability

All data generated or analysed during this study are included in this published article.

## Abbreviations

MEG3: maternally expressed 3
P-STAT3: phosphorylated signal transducer and activator of transcription 3
RIP: RNA-binding protein immunoprecipitation
CHX: cycloheximide
HPV: human papillomavirus
